# Supplementary material for: Microbiota and Postmenopause: The resilience of intestinal bacteria in the face of female hormonal aging
Source: PLoS One. 2025 Jun 18;20(6):e0324712. doi: 10.1371/journal.pone.0324712 (PMC12176181; doi:10.1371/journal.pone.0324712)
Supplement: S1 Table — **Pearson’s correlation with significant association with clinical parameters in post-menopausal women for less than ten years. Parametric data was tested using the Shapiro-Wilk test. **Spearman correlation with significant association with clinical parameters in post-menopausal women for less than ten years. Non-parametric data was tested using the Shapiro-Wilk test. (DOCX) [file pone.0324712.s001.docx]

**Supporting Information**

**S1 Table. Correlation analysis between the ten most abundant bacterial phyla, families, and genera with clinical parameters (Group A n=34 ≤10 YEARS POSTMENOPAUSE)**

| **Group A (n=34)**  **(≤ 10 YEARS POSTMENOPAUSE)** | **Age** | | **Last menstrual**  **cycle** | | **BMI**  **(kg/m^2^)** | |
| --- | --- | --- | --- | --- | --- | --- |
| **Phyla**  **Pearson’s correlation** | *r* | P-value | *r* | P-value | *r* | P-value |
| *Firmicutes* | 0.1441 | 0.4163 | 0.06047 | 0.7341 | -0.1886 | 0.2855 |
| *Bacteroidota* | 0.2325 | 0.1859 | 0.1430 | 0.4197 | -0.2461 | 0.1606 |
| *Proteobacteria* | 0.1682 | 0.3417 | 0.1522 | 0.3903 | -0.2247 | 0.2014 |
| *Actinobacteriota* | 0.1370 | 0.4396 | -0.05527 | 0.7562 | 0.01941 | 0.9132 |
| *Unclassified* | 0.2403 | 0.1710 | 0.01911 | 0.9146 | -0.02976 | 0.8673 |
| *Verrucomicrobiota* | 0.1494 | 0.3991 | -0.1402 | 0.4292 | -0.08544 | 0.6309 |
| *Desulfobacterota* | -0.09948 | 0.5756 | 0.02976 | 0.8673 | -0.1276 | 0.4722 |
| *Fusobacteriota* | 4.618e-005 | 0.9998 | 0.1516 | 0.3921 | -0.02410 | 0.8924 |
| *Cyanobacteria* | -0.03163 | 0.8590 | -0.05348 | 0.7639 | 0.1913 | 0.2784 |
| *Euryarchaeota* | -0.2556 | 0.1446 | -0.1722 | 0.3300 | -0.04035 | 0.8208 |

| **Group A (n=34)**  **(≤ 10 YEARS POSTMENOPAUSE)** | **Age** | | **Last menstrual**  **cycle** | | **BMI**  **(kg/m^2^)** | |
| --- | --- | --- | --- | --- | --- | --- |
| **Families**  **Pearson’s correlation** | *r* | P-value | *r* | P-value | *r* | P-value |
| *Lachnospiraceae* | 0.1228 | 0.4890 | 0.06681 | 0.7074 | -0.2595 | 0.1383 |
| *Bacteroidaceae* | 0.2611 | 0.1358 | 0.03200 | 0.8574 | -0.2571 | 0.1422 |
| *Ruminococcaceae* | 0.09046 | 0.6109 | -0.003468 | 0.9845 | -0.08504 | 0.6325 |
| *Prevotellaceae* | 0.1107 | 0.5331 | 0.1592 | 0.3684 | -0.09116 | 0.6081 |
| *Unclassified* | -0.03731 | 0.8341 | -0.01393 | 0.9377 | -0.06054 | 0.7338 |
| *Veillonellaceae* | 0.1888 | 0.2850 | 0.2084 | 0.2368 | 0.1243 | 0.4835 |
| *Oscillospiraceae* | 0.04069 | 0.8193 | -0.04618 | 0.7954 | -0.2065 | 0.2414 |
| *Christensenellaceae* | -0.04720 | 0.7910 | -0.1595 | 0.3675 | -0.3124 | 0.0720 |
| *Eubacterium_coprostanoligenes_group* | -0.1045 | 0.5562 | 0.1171 | 0.5097 | 0.2236 | 0.2037 |
| *Rikenellaceae* | 0.007397 | 0.9669 | 0.03472 | 0.8454 | -0.3542 | 0.0399* |

| **Group A (n=34)**  **(≤ 10 YEARS POSTMENOPAUSE)** | **Age** | | **Last menstrual**  **cycle** | | **BMI**  **(kg/m^2^)** | |
| --- | --- | --- | --- | --- | --- | --- |
| **Genera**  **Pearson’s correlation** | *r* | P-value | *r* | P-value | *r* | P-value |
| *Bacteroides* | 0.2281 | 0.1946 | 0.05703 | 0.7487 | -0.2169 | 0.2179 |
| *Unclassified* | 0.2403 | 0.1710 | 0.01911 | 0.9146 | -0.02976 | 0.8673 |
| *Prevotella* | 0.1381 | 0.4360 | 0.1917 | 0.2775 | -0.1138 | 0.5218 |
| *Faecalibacterium* | 0.08972 | 0.6139 | 0.06379 | 0.7200 | 0.0003903 | 0.9983 |
| *Agathobacter* | 0.06751 | 0.7044 | 0.02487 | 0.8889 | 0.01766 | 0.9210 |
| *Dialister* | 0.05831 | 0.7433 | 0.05941 | 0.7386 | 0.1550 | 0.3814 |
| *Roseburia* | 0.2615 | 0.1351 | 0.2742 | 0.1165 | -0.01940 | 0.9133 |
| *Subdoligranulum* | 0.1798 | 0.3090 | 0.03026 | 0.8651 | -0.1025 | 0.5641 |
| *Christensenellaceae_R-7_group* | -0.04798 | 0.7876 | -0.1617 | 0.3608 | -0.3129 | 0.0715 |
| *UCG-002* | -0.06506 | 0.7147 | -0.07475 | 0.6744 | -0.1445 | 0.4149 |

**Pearson’s correlation with significant association with clinical parameters in post-menopausal women for less than ten years. Parametric data was tested using the Shapiro-Wilk test.

| **Group A (n=34)**  **(≤ 10 YEARS POSTMENOPAUSE)** | **Post-menopause time (years)** | | **HAS** | | **DM** | | **Osteoporosis** | | **Thyroid diseases** | | **Neuro-psychiatric (Anxiety, Depression or boths)** | |
| --- | --- | --- | --- | --- | --- | --- | --- | --- | --- | --- | --- | --- |
| **Phyla**  **Spermans’s correlation** | *rho* | P-value | *rho* | P-value | *rho* | P-value | *rho* | P-value | *rho* | P-value | *rho* | P-value |
| *Firmicutes* | 0.467 | 0.173 | -0.006 | 0.985 | -0.595 | -0.595 | -0.595 | 0.069 | -0.595 | 0.069 | -0.595 | 0.069 |
| *Bacteroidota* | 0.520 | 0.122 | -0.055 | 0.879 | -0.405 | -0.405 | -0.405 | 0.244 | -0.405 | 0.244 | -0.405 | 0.244 |
| *Proteobacteria* | -0.270 | 0.450 | -0.051 | 0.887 | -0.143 | -0.143 | -0.143 | 0.693 | -0.143 | 0.693 | -0.143 | 0.693 |
| *Actinobacteriota* | 0.398 | 0.254 | 0.044 | 0.903 | -0.411 | -0.411 | -0.411 | 0.237 | -0.411 | 0.237 | -0.411 | 0.237 |
| *Unclassified* | 0.563 | 0.089 | 0.474 | 0.165 | -0.157 | -0.157 | -0.157 | 0.664 | -0.157 | 0.664 | -0.157 | 0.664 |
| *Verrucomicrobiota* | -0.058 | 0.873 | -0.163 | 0.651 | -0.312 | -0.312 | -0.312 | 0.379 | -0.312 | 0.379 | -0.312 | 0.379 |
| *Desulfobacterota* | -0.060 | 0.867 | -0.574 | 0.082 | -0.238 | -0.238 | -0.238 | 0.507 | -0.238 | 0.507 | -0.238 | 0.507 |
| *Fusobacteriota* | 0.330 | 0.3508 | 0.3105 | 0.3825 | 0.0083 | 0.0083 | 0.0083 | 0.9817 | 0.0083 | 0.9817 | 0.0083 | 0.9817 |
| *Cyanobacteria* | -0.6424* | 0.0452* | -0.5034 | 0.1380 | 0.1430 | 0.1430 | 0.1430 | 0.6936 | 0.1430 | 0.6936 | 0.1430 | 0.6936 |
| *Euryarchaeota* | 0.3125 | 0.3794 | 0.3626 | 0.3031 | -0.07760 | -0.07760 | -0.07760 | 0.8313 | -0.0776 | 0.8313 | -0.07760 | 0.8313 |

| **Group A (n=34) (≤ 10 YEARS POSTMENOPAUSE)** | **Post-menopause time (years)** | | **HAS** | | **DM** | | **Osteoporosis** | | **Thyroid diseases** | | **Neuro-psychiatric (Anxiety, Depression, or both)** | |
| --- | --- | --- | --- | --- | --- | --- | --- | --- | --- | --- | --- | --- |
| **Families**  **Spermans’s correlation** | *rho* | P-value | *rho* | P-value | *rho* | P-value | *rho* | P-value | *rho* | P-value | *rho* | P-value |
| *Lachnospiraceae* | 0.01832 | 0.9181 | -0.1203 | 0.4979 | -0.003707 | 0.9834 | 0.3350 | 0.0528 | 0.2435 | 0.1653 | -0.3058 | 0.0786 |
| *Bacteroidaceae* | 0.2721 | 0.1196 | -0.1820 | 0.3029 | 0.03337 | 0.8514 | 0.8514 | 0.2242 | 0.2435 | 0.1653 | 0.05097 | 0.7747 |
| *Ruminococcaceae* | 0.04449 | 0.8027 | 0.01542 | 0.9310 | 0.05561 | 0.7548 | 0.4280 | 0.0116* | 0.1974 | 0.2632 | -0.2752 | 0.1152 |
| *Prevotellaceae* | -0.04413 | 0.8043 | 0.1401 | 0.4294 | 0.1833 | 0.2993 | -0.009391 | 0.9580 | 0.01992 | 0.9110 | -0.1817 | 0.3036 |
| *Unclassified* | -0.01018 | 0.9545 | -0.02378 | 0.8938 | -0.04900 | 0.7832 | 0.1999 | 0.2571 | 0.1594 | 0.3677 | -0.04491 | 0.8009 |
| *Veillonellaceae* | -0.00728 | 0.9674 | 0.5091 | 0.0021** | 0.1492 | 0.3995 | 0.1077 | 0.5443 | 0.2682 | 0.1251 | -0.1436 | 0.4177 |
| *Oscillospiraceae* | -0.04095 | 0.8181 | -0.02776 | 0.8762 | -0.02595 | 0.8842 | 0.1024 | 0.5646 | 0.1513 | 0.3929 | -0.09174 | 0.6059 |
| *Christensenellaceae* | -0.1594 | 0.3679 | -0.2443 | 0.1637 | -0.01859 | 0.9169 | -0.05131 | 0.7732 | -0.0362 | 0.8386 | -0.1601 | 0.3657 |
| *Eubacterium_coprostanoligenes_group* | -0.2294 | 0.1918 | 0.2070 | 0.2402 | 0.3527 | 0.0408* | -0.04660 | 0.7936 | -0.0527 | 0.7672 | -0.1327 | 0.4543 |
| *Rikenellaceae* | -0.02683 | 0.8803 | -0.1205 | 0.4973 | -0.03342 | 0.8512 | 0.1491 | 0.4000 | 0.07908 | 0.6567 | -0.2484 | 0.1566 |

| **Group A (n=34) (≤ 10 YEARS POSTMENOPAUSE)** | **Post-menopause time (years)** | | **HAS** | | **DM** | | **Osteoporosis** | | **Thyroid diseases** | | **Neuro-psychiatric (Anxiety, Depression, or both)** | |
| --- | --- | --- | --- | --- | --- | --- | --- | --- | --- | --- | --- | --- |
| **Genera**  **Spermans’s correlation** | *rho* | P-value | *rho* | P-value | *rho* | P-value | *rho* | P-value | *rho* | P-value | *rho* | P-value |
| *Bacteroides* | 0.1523 | 0.3899 | -0.2684 | 0.1249 | -0.1149 | 0.5175 | 0.2326 | 0.1855 | 0.1316 | 0.4582 | 0.08495 | 0.6329 |
| *Unclassified* | -0.03737 | 0.8338 | 0.1120 | 0.5283 | -0.1396 | 0.4311 | 0.04380 | 0.8057 | -0.0353 | 0.8425 | -0.01371 | 0.9387 |
| *Prevotella* | -0.06892 | 0.6986 | 0.1238 | 0.4855 | 0.02670 | 0.8808 | -0.06702 | 0.7065 | 0.06770 | 0.7036 | -0.1573 | 0.3742 |
| *Faecalibacterium* | 0.05164 | 0.7718 | 0.08660 | 0.6263 | 0.1375 | 0.4380 | 0.3639 | 0.0344* | 0.1814 | 0.3045 | -0.2044 | 0.2462 |
| *Agathobacter* | 0.0004626 | 0.9979 | 0.08959 | 0.6144 | 0.1374 | 0.4385 | 0.3075 | 0.0768 | 0.1450 | 0.4133 | -0.1599 | 0.3662 |
| *Dialister* | 0.1352 | 0.4458 | 0.4803 | 0.0040** | 0.07060 | 0.6915 | 0.09382 | 0.5977 | 0.1290 | 0.4672 | -0.03045 | 0.8643 |
| *Roseburia* | 0.01340 | 0.9400 | -0.03105 | 0.8616 | 0.07462 | 0.6749 | 0.07960 | 0.6545 | 0.1192 | 0.5019 | -0.09917 | 0.5768 |
| *Subdoligranulum* | 0.07386 | 0.6781 | -0.009268 | 0.9585 | 0.06312 | 0.7229 | 0.3821 | 0.0258* | 0.2504 | 0.1532 | -0.2212 | 0.2087 |
| *Christensenellaceae_R-7_group* | -0.1463 | 0.4091 | -0.2320 | 0.1868 | -0.01115 | 0.9501 | -0.04198 | 0.8136 | -0.0296 | 0.8676 | -0.1669 | 0.3454 |
| *UCG-002* | -0.09681 | 0.5860 | 0.07783 | 0.6617 | 0.2058 | 0.2430 | 0.04696 | 0.7920 | -0.0066 | 0.9703 | -0.09945 | 0.5758 |

**Spearman correlation with significant association with clinical parameters in post-menopausal women for less than ten years. Non-parametric data was tested using the Shapiro-Wilk test.
