# Supplementary material for: Microbiota and Postmenopause: The resilience of intestinal bacteria in the face of female hormonal aging
Source: PLoS One. 2025 Jun 18;20(6):e0324712. doi: 10.1371/journal.pone.0324712 (PMC12176181; doi:10.1371/journal.pone.0324712)
Supplement: S2 Table — **Pearson’s correlation with significant association with clinical parameters in post-menopausal women for more than ten years. Parametric data was tested using the Shapiro-Wilk test. ** Spearman’s correlation with significant association with clinical parameters in post-menopausal women for more than ten years. Parametric data was tested using the Shapiro-Wilk test. (DOCX) [file pone.0324712.s002.docx]

**Supporting Information**

**S2 Table. Correlation analysis between the ten most abundant bacterial phyla, families, and genera with clinical parameters (Group B n=10 ≥ 10 YEARS POSTMENOPAUSE)**

| **Group B (n=10)**  **(≥ 10 YEARS POSTMENOPAUSE)** | **Age** | | **Last menstrual**  **cycle** | | **BMI**  **(kg/m^2^)** | |
| --- | --- | --- | --- | --- | --- | --- |
| **Phyla**  **Pearson’s correlation** | *r* | P-value | *r* | P-value | *r* | P-value |
| *Firmicutes* | 0.467 | 0.173 | -0.006 | 0.985 | -0.595 | 0.069 |
| *Bacteroidota* | 0.520 | 0.122 | -0.055 | 0.879 | -0.405 | 0.244 |
| *Proteobacteria* | -0.270 | 0.450 | -0.051 | 0.887 | -0.143 | 0.693 |
| *Actinobacteriota* | 0.398 | 0.254 | 0.044 | 0.903 | -0.411 | 0.237 |
| *Unclassified* | 0.563 | 0.089 | 0.474 | 0.165 | -0.157 | 0.664 |
| *Verrucomicrobiota* | -0.058 | 0.873 | -0.163 | 0.651 | -0.312 | 0.379 |
| *Desulfobacterota* | -0.060 | 0.867 | -0.574 | 0.082 | -0.238 | 0.507 |
| *Fusobacteriota* | 0.330 | 0.3508 | 0.3105 | 0.3825 | 0.0083 | 0.9817 |
| *Cyanobacteria* | -0.6424* | 0.0452* | -0.5034 | 0.1380 | 0.1430 | 0.6936 |
| *Euryarchaeota* | 0.3125 | 0.3794 | 0.3626 | 0.3031 | -0.07760 | 0.8313 |

| **Group B (n=10)**  **(≥ 10 YEARS POSTMENOPAUSE)** | **Age** | | **Last menstrual**  **cycle** | | **BMI**  **(kg/m^2^)** | |
| --- | --- | --- | --- | --- | --- | --- |
| **Families**  **Pearson’s correlation** | *r* | P-value | *r* | P-value | *r* | P-value |
| *Lachnospiraceae* | 0.3189 | 0.3692 | -0.2267 | 0.5288 | -0.6236 | 0.0541 |
| *Bacteroidaceae* | 0.4690 | 0.1715 | -0.09065 | 0.8033 | -0.4033 | 0.2478 |
| *Ruminococcaceae* | 0.1536 | 0.6718 | -0.07732 | 0.8319 | -0.4115 | 0.2374 |
| *Prevotellaceae* | -0.7068 | 0.0223* | -0.5425 | 0.1052 | 0.2607 | 0.4670 |
| *Unclassified* | 0.5763 | 0.0812 | 0.4889 | 0.1516 | -0.1665 | 0.6457 |
| *Veillonellaceae* | -0.5809 | 0.0782 | -0.1771 | 0.6246 | 0.01336 | 0.9708 |
| *Oscillospiraceae* | 0.2810 | 0.4316 | 0.07923 | 0.8278 | -0.03015 | 0.9341 |
| *Christensenellaceae* | -0.01288 | 0.9718 | -0.2557 | 0.4758 | 0.03765 | 0.9178 |
| *Eubacterium_coprostanoligenes_group* | -0.4119 | 0.2370 | -0.5442 | 0.1039 | -0.02815 | 0.9385 |
| *Rikenellaceae* | 0.3297 | 0.3522 | -0.1387 | 0.7024 | -0.3220 | 0.3642 |

| **Group B (n=10)**  **(≥ 10 YEARS POSTMENOPAUSE)** | **Age** | | **Last menstrual**  **cycle** | | **BMI**  **(kg/m^2^)** | |
| --- | --- | --- | --- | --- | --- | --- |
| **Genera**  **Pearson’s correlation** | *r* | P-value | *r* | P-value | *r* | P-value |
| *Bacteroides* | 0.4690 | 0.1715 | -0.09065 | 0.8033 | -0.4033 | 0.2478 |
| *Unclassified* | 0.5763 | 0.082 | 0.4889 | 0.1516 | -0.1665 | 0.6457 |
| *Prevotella* | -0.6160 | 0.0579 | -0.5259 | 0.1185 | 0.1758 | 0.6271 |
| *Faecalibacterium* | -0.06990 | 0.8478 | -0.08149 | 0.8229 | -0.1216 | 0.7378 |
| *Agathobacter* | 0.3005 | 0.3989 | 0.1375 | 0.7048 | -0.5741 | 0.0826 |
| *Dialister* | -0.5960 | 0.0690 | -0.1932 | 0.5927 | 0.01319 | 0.9711 |
| *Roseburia* | -0.2249 | 0.5322 | -0.6323 | 0.0498* | -0.1423 | 0.6950 |
| *Subdoligranulum* | -0.08125 | 0.8234 | -0.4812 | 0.1591 | -0.4268 | 0.2187 |
| *Christensenellaceae_R-7_group* | -0.04115 | 0.9101 | -0.2527 | 0.4812 | 0.07073 | 0.8460 |
| *UCG-002* | -0.7115 | 0.0210* | -0.7819 | 0.0075** | 0.4787 | 0.1616 |

**Pearson’s correlation with significant association with clinical parameters in post-menopausal women for more than ten years. Parametric data was tested using the Shapiro-Wilk test.

| **Group B (n=10)**  **(≥ 10 YEARS POSTMENOPAUSE)** | **Post-menopause time (years)** | | **HAS** | | **DM** | | **Osteoporosis** | | **Thyroid diseases** | | **Neuro-psychiatric (Anxiety, Depression, or both)** | |
| --- | --- | --- | --- | --- | --- | --- | --- | --- | --- | --- | --- | --- |
| **Phyla**  **Spermans’s correlation** | *rho* | P-value | *rho* | P-value | *rho* | P-value | *rho* | P-value | *rho* | P-value | *rho* | P-value |
| *Firmicutes* | 0.2339 | 0.5137 | -0.6458 | 0.0667 | -0.6093 | 0.0889 | 0.08704 | 0.8889 | 0.3419 | 0.3833 | 0.5685 | 0.1143 |
| *Bacteroidota* | 0.4000 | 0.2511 | -0.0379 | >0.9999 | 0.08704 | 0.8889 | 0.4352 | 0.2667 | -0.3419 | 0.3833 | 0.000 | >0.9999 |
| *Proteobacteria* | -0.04924 | 0.8978 | -0.4938 | 0.1833 | -0.5222 | 0.1778 | -0.08704 | 0.8889 | 0.4179 | 0.2667 | 0.2132 | 0.6095 |
| *Actinobacteriota* | 0.2277 | 0.5243 | -0.0379 | >0.9999 | 0.2611 | 0.5333 | 0.4352 | 0.2667 | -0.3419 | 0.3833 | 0.3553 | 0.3524 |
| *Unclassified* | -0.1318 | 0.7155 | -0.3253 | 0.4167 | -0.1398 | 0.7778 | 0.5124 | 0.1111 | 0.2440 | 0.5417 | -0.03804 | >0.9999 |
| *Verrucomicrobiota* | 0.2928 | 0.4085 | 0.03846 | >0.9999 | 0.3525 | 0.4000 | -0.08811 | 0.8889 | -0.1923 | 0.6667 | 0.5036 | 0.1714 |
| *Desulfobacterota* | 0.5293 | 0.1189 | -0.4938 | 0.1833 | -0.2611 | 0.5333 | -0.1741 | 0.7111 | -0.1899 | 0.6667 | 0.2843 | 0.4762 |
| *Fusobacteriota* | -0.05893 | >0.9999 | 0.5092 | 0.3000 | -0.1667 | >0.9999 | -0.1667 | >0.9999 | 0.5092 | 0.3000 | -0.2722 | >0.9999 |
| *Cyanobacteria* | 0.1596 | 0.6597 | -0.4097 | 0.2396 | -0.3022 | 0.3961 | -0.3022 | 0.3961 | 0.2087 | 0.5628 | 0.4729 | 0.1675 |
| *Euryarchaeota* | -0.4333 | 0.2138 | -0.2837 | 0.5000 | -0.09285 | 0.8889 | 0.1857 | 0.6667 | 0.3647 | 0.3333 | 0.07581 | 0.9048 |

| **Group B (n=10)**  **(≥ 10 YEARS POSTMENOPAUSE)** | **Post-menopause time (years)** | | **HAS** | | **DM** | | **Osteoporosis** | | **Thyroid diseases** | | **Neuro-psychiatric (Anxiety, Depression, or both)** | |
| --- | --- | --- | --- | --- | --- | --- | --- | --- | --- | --- | --- | --- |
| **Families**  **Spermans’s correlation** | *rho* | P-value | *rho* | P-value | *rho* | P-value | *rho* | P-value | *rho* | P-value | *rho* | P-value |
| *Lachnospiraceae* | 0.01832 | 0.9181 | -0.1203 | 0.4979 | -0.003707 | 0.9834 | 0.3350 | 0.0528 | 0.2435 | 0.1653 | -0.3058 | 0.0786 |
| *Bacteroidaceae* | 0.2721 | 0.1196 | -0.1820 | 0.3029 | 0.03337 | 0.8514 | 0.2140 | 0.2242 | 0.2435 | 0.1653 | 0.05097 | 0.7747 |
| *Ruminococcaceae* | 0.04449 | 0.8027 | 0.01542 | 0.9310 | 0.05561 | 0.7548 | 0.4280 | 0.0116* | 0.1974 | 0.2632 | -0.2752 | 0.1152 |
| *Prevotellaceae* | -0.04413 | 0.8043 | 0.1401 | 0.4294 | 0.1833 | 0.2993 | -0.009391 | 0.9580 | 0.01992 | 0.9110 | -0.1817 | 0.3036 |
| *Unclassified* | -0.01018 | 0.9545 | -0.02378 | 0.8938 | -0.04900 | 0.7832 | 0.1999 | 0.2571 | 0.1594 | 0.3677 | -0.04491 | 0.8009 |
| *Veillonellaceae* | -0.00728 | 0.9674 | 0.5091 | 0.0021** | 0.1492 | 0.3995 | 0.1077 | 0.5443 | 0.2682 | 0.1251 | -0.1436 | 0.4177 |
| *Oscillospiraceae* | -0.04095 | 0.8181 | -0.02776 | 0.8762 | -0.02595 | 0.8842 | 0.1024 | 0.5646 | 0.1513 | 0.3929 | -0.09174 | 0.6059 |
| *Christensenellaceae* | -0.1594 | 0.3679 | -0.2443 | 0.1637 | -0.01859 | 0.9169 | -0.05131 | 0.7732 | -0.0362 | 0.8386 | -0.1601 | 0.3657 |
| *Eubacterium_coprostanoligenes_group* | -0.2294 | 0.1918 | 0.2070 | 0.2402 | 0.3527 | 0.0408* | -0.04660 | 0.7936 | -0.0527 | 0.7672 | -0.1327 | 0.4543 |
| *Rikenellaceae* | -0.02683 | 0.8883 | -0.1205 | 0.4973 | -0.03342 | 0.8512 | 0.1491 | 0.4000 | 0.07908 | 0.6567 | -0.2484 | 0.1566 |

| **Group B (n=10)**  **(≥ 10 YEARS POSTMENOPAUSE)** | **Post-menopause time (years)** | | **HAS** | | **DM** | | **Osteoporosis** | | **Thyroid diseases** | | **Neuro-psychiatric (Anxiety, Depression, or both)** | |
| --- | --- | --- | --- | --- | --- | --- | --- | --- | --- | --- | --- | --- |
| **Genera**  **Spermans’s correlation** | *rho* | P-value | *rho* | P-value | *rho* | P-value | *rho* | P-value | *rho* | P-value | *rho* | P-value |
| *Bacteroides* | 0.6462 | 0.0485* | 0.1899 | 0.6667 | 0.1741 | 0.7111 | 0.000 | >0.9999 | -0.5698 | 0.1167 | 0.07107 | 0.9143 |
| *Unclassified* | 0.1351 | 0.7033 | -0.6099 | 0.1250 | -0.4658 | 0.3333 | 0.1863 | 0.5556 | 0.2440 | 0.5417 | 0.2282 | 0.5952 |
| *Prevotella* | 0.08667 | 0.8044 | -0.5136 | 0.2500 | -0.3922 | 0.4667 | -0.3922 | 0.4667 | -0.0428 | >0.9999 | 0.3203 | 0.3571 |
| *Faecalibacterium* | 0.3693 | 0.2927 | 0.1140 | 0.8333 | -0.2611 | 0.5333 | -0.3482 | 0.4000 | 0.2659 | 0.5167 | 0.4975 | 0.1714 |
| *Agathobacter* | 0.09232 | 0.8008 | 0.03799 | >0.9999 | 0.000 | >0.9999 | 0.08704 | 0.8889 | -0.1899 | 0.6667 | 0.5685 | 0.1143 |
| *Dialister* | -0.3848 | 0.2657 | -0.5136 | 0.2500 | -0.3922 | 0.4667 | -0.04903 | >0.9999 | 0.3424 | 0.4000 | 0.3203 | 0.3571 |
| *Roseburia* | 0.6462 | 0.0485* | -0.1140 | 0.8333 | -0.2611 | 0.5333 | -0.3482 | 0.4000 | -0.0379 | >0.9999 | 0.1421 | 0.7619 |
| *Subdoligranulum* | 0.5896 | 0.0764 | -0.3810 | 0.2833 | -0.4802 | 0.2000 | -0.6984 | 0.0444* | -0.1524 | 0.7167 | 0.5703 | 0.1095 |
| *Christensenellaceae_R-7_group* | -0.2093 | 0.5608 | -0.5698 | 0.1167 | -0.3482 | 0.4000 | 0.000 | >0.9999 | 0.2659 | 0.5167 | -0.1421 | 0.7619 |
| *UCG-002* | 0.2284 | 0.5201 | -0.03810 | 0.9500 | 0.08730 | 0.8444 | -0.3929 | 0.2889 | -0.1524 | 0.7167 | -0.03564 | 0.9714 |

** Spearman’s correlation with significant association with clinical parameters in post-menopausal women for more than ten years. Parametric data was tested using the Shapiro-Wilk test.
