# Supplementary material for: Microbiota and Postmenopause: The resilience of intestinal bacteria in the face of female hormonal aging
Source: PLoS One. 2025 Jun 18;20(6):e0324712. doi: 10.1371/journal.pone.0324712 (PMC12176181; doi:10.1371/journal.pone.0324712)
Supplement: Attachment — Submitted filename: Response to Reviewers.docx [file pone.0324712.s004.docx]

**Dear Dr. Sayed Haidar Abbas Raza**

Regarding the manuscript [PONE-D-24-55161] - [EMID:5576289f144cdfbc] entitled “Microbiota and Postmenopause: The Resilience of Intestinal Bacteria in the Face of Female Hormonal Aging,” we have carefully reviewed and revised the manuscript according to the reviewers' suggestions. Below, we address each point raised, indicating where changes were made in the text. We would like to take this opportunity to sincerely thank the reviewers for their thorough and insightful feedback, which greatly facilitated our revision process and undoubtedly enhanced the quality of our manuscript.

1. **Please ensure that your manuscript meets PLOS ONE's style requirements, including those for file naming.**

The manuscript was checked for PLOS ONE style requirements, titles, subtitles, symbols and file nomenclature.

2. **Please amend either the title on the online submission form (via Edit Submission) or the title in the manuscript so that they are identical.**

The title on the online submission form and the manuscript have been revised.

3. **We note that the grant information you provided in the ‘Funding Information’ and ‘Financial Disclosure’ sections do not match. When you resubmit, please ensure that you provide the correct grant numbers for the awards you received for your study in the ‘Funding Information’ section.**

The Financing and Financial Disclosure information has been revised.

4. **Thank you for stating the following financial disclosure: [This research is supported by CAPES (Coordenação de Aperfeiçoamento de Pessoal de Nível Superior), CNPQ (Conselho Nacional de Desenvolvimento Científico e Tecnológico), Rede Minas Microbioma, Projeto RED-00181-23 FAPEMIG, and the Brazilian National Council for Scientific and Technological Development.]. Please state what role the funders took in the study. If the funders had no role, please state: "The funders had no role in study design, data collection and analysis, decision to publish, or preparation of the manuscript." If this statement is not correct you must amend it as needed. Please include this amended Role of Funder statement in your cover letter; we will change the online submission form on your behalf.**

This study was funded in part by CAPES (Coordination for the Improvement of Higher Education Personnel - Brazil) - Funding code 001. By the Rede Minas Microbioma, Project RED-00132-16 FAPEMIG and by CNPq (Conselho Nacional de Desenvolvimento Científico e Tecnológico).

5. **We note that you have indicated that there are restrictions to data sharing for this study. PLOS only allows data to be available upon request if there are legal or ethical restrictions on sharing data publicly.**

**a) If there are ethical or legal restrictions on sharing a de-identified data set, please explain them in detail (e.g., data contain potentially identifying or sensitive patient information, data are owned by a third-party organization, etc.) and who has imposed them (e.g., a Research Ethics Committee or Institutional Review Board, etc.). Please also provide contact information for a data access committee, ethics committee, or other institutional body to which data requests may be sent.**

**b) If there are no restrictions, please upload the minimal anonymized data set necessary to replicate your study findings to a stable, public repository and provide us with the relevant URLs, DOIs, or accession numbers. For a list of recommended repositories, please see https://journals.plos.org/plosone/s/recommended-repositories. You also have the option of uploading the data as Supporting Information files, but we would recommend depositing data directly to a data repository if possible.**

A dataset and workflow can be found at the following public domain address: <https://github.com/bioinfonupeb/redemicro-thayane>

The sequencing data is deposited at NCBI under the BioProject ID PRJNA1223961 <http://www.ncbi.nlm.nih.gov/bioproject/1223961>

6. **In the online submission form, you indicated that [The data is available. Contact corresponding author Renata Guerra-Sá email** [**rguerra@ufop.edu.br**](mailto:rguerra@ufop.edu.br)**].  All PLOS journals now require all data underlying the findings described in their manuscript to be freely available to other researchers, either 1. In a public repository, 2. Within the manuscript itself, or 3. Uploaded as supplementary information. This policy applies to all data except where public deposition would breach compliance with the protocol approved by your research ethics board. If your data cannot be made publicly available for ethical or legal reasons (e.g., public availability would compromise patient privacy), please explain your reasons on resubmission and your exemption request will be escalated for approval.**

A dataset and workflow can be found at the following public domain address: <https://github.com/bioinfonupeb/redemicro-thayane>

The sequencing data is deposited at NCBI under the BioProject ID PRJNA1223961 <http://www.ncbi.nlm.nih.gov/bioproject/1223961>

In addition to this, other important data has been made available as supplementary information.

7. **Please include captions for your Supporting Information files at the end of your manuscript, and update any in-text citations to match accordingly. Please see our Supporting Information guidelines for more information:**

Captions have been included in the supporting information files, figures and tables. The order of citation in the text has also been checked.

**ANSWER TO REVIEWER n°1**

**1. While the article notes that some participants used antihypertensive, antidiabetic, and other medications, there is no in-depth discussion of how these confounders might have influenced the gut microbiota composition. More elaboration on how such factors were controlled or factored into the analysis would strengthen the study.**

**Answer:** We acknowledge the importance of antihypertensive and antidiabetic drugs in shaping gut microbiome composition. While these factors were recorded as potential confounders, they were not directly included in the adjusted analyses due to the limited availability of data stratified by specific medications. For future studies, we recommend incorporating a control group with similar characteristics but without medication use, as well as conducting additional analyses to better assess the potential impact of these drugs on microbial diversity.

**Please see lines 478-493:** Critical reviews show that the use of antidiabetic and antihypertensive drugs acts to modify and regulate the intestinal microbiota (65,71,72). Firmicutes and Proteobacteria are enriched with the use of Captopril (65). At the same time, metformin is primarily related to the increase in genera such as Prevotella (68) and the enrichment of Akkermansia muciniphila, which reflects beneficial mechanisms such as the production of short-chain fatty acids by this bacterium, regulation of glycemic indices and protection and integrity of the gastrointestinal tract (73). In contrast, metformin reduced the population of Roseburia and Faecalibacterium (74). Antidepressants also affect intestinal bacteria, modifying the concentration of secondary metabolites responsible for gut-brain communication (75).

Based on the literature already published, our research group recognizes the importance and relevance of the impact of drugs such as antihypertensives and antidiabetics on the composition of the gut microbiome. In this Brazilian cohort, these factors were recorded as potential confounding factors. Still, they were not directly included in the adjusted analyses due to the limited data stratified by specific drugs. For future studies, we recommend including a control group with similar characteristics but without medications and additional analyses to assess the possible influences of these medications on microbial diversity.

**Please see lines 521-539:** Our research has significant limitations that must be reported when studying gut microbiota. We recognize the limitation of a smaller sample size (n=10) in the postmenopausal group for more than 10 years. The small sample size reduces the statistical power to identify subtle differences in microbial composition. Future studies with more participants in this category will be essential to validate our initial observations and give greater robustness to the conclusions. It was not possible to investigate and monitor dietary habits and practice of physical activities. The participants reported using different classes of medication, except antibiotics. We cannot rule out the possibility that these factors may influence the composition of these participants' microbiota. Future studies should be carried out to control these factors in this cohort, and predictive functional analysis should also be included to strengthen the characterization of the intestinal microbiota of Brazilian women.

The strengths of our study involve the participation of a cohort of postmenopausal women whose age range coincides with significant hormonal and metabolic changes primarily related to essential modifications in the intestinal microbiota. Our study contributes to the characterization of this microbiota in middle-aged Brazilian women, showing their resilience, which may open the way for individualized and personalized treatments for conditions that affect women at this stage of life. In addition, we characterized the ten most abundant organisms in this population, which could indicate new actions to prevent postmenopausal health conditions.

**2.With only 10 women in the group that have been postmenopausal for more than 10 years, the statistical power to detect meaningful differences is somewhat limited. This may question whether sample size restrictions impacted the study’s ability to detect subtler microbiome shifts.**

**Answer:** We acknowledge the limitation of a smaller sample size (n=10) in the group of postmenopausal women for more than 10 years, which may reduce the statistical power to detect subtle differences in microbial composition. However, despite this limitation, our results remain relevant, as there are few studies focusing on Brazilian cohorts. Future studies with a larger number of participants in this category will be essential to validate our initial findings and strengthen the robustness of our conclusions.

**Please see lines 521-539:** Our research has significant limitations that must be reported when studying gut microbiota. We recognize the limitation of a smaller sample size (n=10) in the postmenopausal group for more than 10 years. The small sample size reduces the statistical power to identify subtle differences in microbial composition. Future studies with more participants in this category will be essential to validate our initial observations and give greater robustness to the conclusions. It was not possible to investigate and monitor dietary habits and practice of physical activities. The participants reported using different classes of medication, except antibiotics. We cannot rule out the possibility that these factors may influence the composition of these participants' microbiota. Future studies should be carried out to control these factors in this cohort, and predictive functional analysis should also be included to strengthen the characterization of the intestinal microbiota of Brazilian women.

The strengths of our study involve the participation of a cohort of postmenopausal women whose age range coincides with significant hormonal and metabolic changes primarily related to essential modifications in the intestinal microbiota. Our study contributes to the characterization of this microbiota in middle-aged Brazilian women, showing their resilience, which may open the way for individualized and personalized treatments for conditions that affect women at this stage of life. In addition, we characterized the ten most abundant organisms in this population, which could indicate new actions to prevent postmenopausal health conditions.

**3. While alpha and beta diversity metrics and specific statistical tests (Kruskal-Wallis, PERMANOVA, etc.) are mentioned, the text does not elaborate on multiple testing corrections or whether the p-values were adjusted for multiple comparisons. Clarifying these points would add rigor to the findings.**

**Answer:** Although alpha and beta diversity analyses were conducted using PERMANOVA and Kruskal-Wallis tests, we acknowledge the importance of explicitly addressing multiple testing corrections. To mitigate false positives, we will apply standard corrections, such as the Benjamini-Hochberg method, and clarify these details in the text to enhance transparency and methodological rigor.

**Please see lines 164-165:** The Benjamini-Hochberg correction method was used to adjust the p-values.

**Minor Mistakes**

**1. Occasional double “Note: Note:” statements and extra spaces around parentheses can distract readers. A careful proofreading for typographical errors (e.g., extraneous spacing, repeated words) would improve readability.**

**Answer:** We thank you for your comment and will review the manuscript thoroughly to correct typographical errors, such as repetitions of 'Note: Note:' and unnecessary spacing. This effort will also include eliminating redundancies in the sections related to aging, hormonal decline, and changes in the microbiome, consolidating the information for better clarity.

**Please see lines 01-931:** All text has been revised.

**2. The manuscript mentions figures and tables (Figure 3, Table 2, etc.) that are presumably provided, but some figure or table references might appear out of order or without a fully explanatory caption. Ensuring the figures and tables are clearly labeled, cited in the correct order, and have complete legends will help the flow of the text.**

**Answer:** We thoroughly checked the figures and tables to ensure that they were all cited in the correct order and with full explanatory captions. This improvement will help maintain the flow of the text and make it easier for the reader to understand. All figures and tables are clearly labeled, with a title and explanatory caption, and cited in the correct order in the text.

**Please see lines 182-188: Table 1. Sociodemographic and health characteristics of the women participating in this study.**

**Please see lines 200-203: Table 2. Data inferred after quality control and merging reads obtained for single-end and paired-end.**

**Please see lines 311-315: Table 3. Taxonomic inference was found in each group according to the time after menopause.**

**Please see lines 205-209: Fig 1. Rarefaction curve of the features observed per sample analyzed (n=44).**

**Please see lines 224-240: Fig 2. Alpha diversity of the fecal microbiota presented by the Shannon and Simpson index.**

**Please see lines 248-258:** **Fig 3. Unweighted principal coordinate analysis (PCoA) of the bacterial communities presents in the fecal microbiota of postmenopausal women.**

**Please see lines 260-278:** **Fig 4. Analysis of the fecal microbiota's beta diversity according to post menopause time.**

**Please see lines 281-301: Fig 5. Relative abundance analysis at Phylum level (a), Family level (b) and Genus level (c) based on 16S rRNA data.**

**Please see lines 335-342: Fig 6. Classification of the 10 most abundant phyla according to their relative abundance by groups.**

**Please see lines 344-351:** **Fig 7. Classification of the 10 most abundant Family according to their relative abundance by groups.**

**Please see lines 353-359: Fig 8. Classification of the 10 most abundant Genera according to their relative abundance by groups.**

**Please see lines 952-996 Supplementary material: S1 Table**. **Medications used by participants in this study.**

**Please see lines 952-996 Supplementary material: S2 Table. Correlation analysis between the ten most abundant bacterial phyla, families and genera with clinical parameters**

**3. In a few paragraphs, ideas about aging, hormone decline, and microbiota changes are repeated in very similar wording. More concise phrasing or combining those sentences under the same subheading would avoid redundancy.**

**Answer:** Thank you for your comment. We have reviewed the terms indicated and modified them for a more concise reading.

**4. Some terms like “p-value,” “F-value,” or how certain tests were run (e.g., “999 permutations”) could be formatted consistently across all results sections. A small but notable improvement would be using standard statistical reporting guidelines uniformly.**

**Answer:** We agree that consistency in formatting statistical terms, such as 'p-value' and 'F-value,' is fundamental. We will adjust to following standardized statistical reporting guidelines, using uniform formatting throughout the manuscript.

**Please see lines 275-278:** Legend: Group A n=34 (≤10 years postmenopause); Group B n=10 (≥10 years postmenopause). A) The ANOSIM (test R = -0.171; p-value = 0.98; N.Perm = 999). B) The statistical significance was assessed using permutational multivariate analysis of variance (PERMANOVA) (Pseudo-F = 0.972; p-value:0.492; N.Perm = 999).

**ANSWER TO REVIEWER n°2**

**1. The study does not adequately control for confounding factors such as diet, physical activity, and medication use. These factors can significantly influence the gut microbiome composition. The authors should address how these variables were accounted for in their analysis or discuss them as limitations.**

**Answer:** Thank you for your valuable feedback. While we did not directly adjust for factors such as diet, physical activity, and medication use, these variables were recorded during data collection and qualitatively analyzed. We acknowledge that incorporating them as covariates in future analyses would strengthen the study. Additionally, we will emphasize these limitations in the discussion, recognizing their potential influence on gut microbiome composition.

**Please see lines 464-468:** Numerous disturbances expose the microbiome throughout its host's life. The type of diet consumed, physical exercise or a sedentary lifestyle, infections, and the use of medication, particularly antibiotics, are widely reported as disturbances that can lead to unhealthy states of the microbiome and, depending on the degree of this bacterial disturbance, can be referred to as dysbiosis (63,64).

**Please see lines 478-493:** Critical reviews show that the use of antidiabetic and antihypertensive drugs acts to modify and regulate the intestinal microbiota (65,71,72). Firmicutes and Proteobacteria are enriched with the use of Captopril (65). At the same time, metformin is primarily related to the increase in genera such as Prevotella (68) and the enrichment of Akkermansia muciniphila, which reflects beneficial mechanisms such as the production of short-chain fatty acids by this bacterium, regulation of glycemic indices and protection and integrity of the gastrointestinal tract (73). In contrast, metformin reduced the population of Roseburia and Faecalibacterium (74). Antidepressants also affect intestinal bacteria, modifying the concentration of secondary metabolites responsible for gut-brain communication (75).

Based on the literature already published, our research group recognizes the importance and relevance of the impact of drugs such as antihypertensives and antidiabetics on the composition of the gut microbiome. In this Brazilian cohort, these factors were recorded as potential confounding factors. Still, they were not directly included in the adjusted analyses due to the limited data stratified by specific drugs. For future studies, we recommend including a control group with similar characteristics but without medications and additional analyses to assess the possible influences of these medications on microbial diversity.

**Please see lines 497-504:** A pilot study conducted in our laboratory in 2024 (unpublished data) used qPCR to evaluate the intestinal microbiota of postmenopausal women using hypoglycemic, antihypertensive, and lipid-lowering medications and those who were not using such medications. The genera *Akkermansia, Roseburia*, and *Faecalibacterium prausnitzii* were quantified, and no differences were found in the quantification of these organisms among the participants. This pilot study corroborates the sequencing findings (Figure 8), which also showed no differences for *Roseburia* and *Faecalibacterium prausnitzii*, indicating that such medications did not cause significant changes in these organisms in this cohort.

**Please see lines 521-539:** Our research has significant limitations that must be reported when studying gut microbiota. We recognize the limitation of a smaller sample size (n=10) in the postmenopausal group for more than 10 years. The small sample size reduces the statistical power to identify subtle differences in microbial composition. Future studies with more participants in this category will be essential to validate our initial observations and give greater robustness to the conclusions. It was not possible to investigate and monitor dietary habits and practice of physical activities. The participants reported using different classes of medication, except antibiotics. We cannot rule out the possibility that these factors may influence the composition of these participants' microbiota. Future studies should be carried out to control these factors in this cohort, and predictive functional analysis should also be included to strengthen the characterization of the intestinal microbiota of Brazilian women.

The strengths of our study involve the participation of a cohort of postmenopausal women whose age range coincides with significant hormonal and metabolic changes primarily related to essential modifications in the intestinal microbiota. Our study contributes to the characterization of this microbiota in middle-aged Brazilian women, showing their resilience, which may open the way for individualized and personalized treatments for conditions that affect women at this stage of life. In addition, we characterized the ten most abundant organisms in this population, which could indicate new actions to prevent postmenopausal health conditions.

**2. The sequencing methods differed for some samples (38 paired-end vs. 6 single-end). The authors should explain how this discrepancy might affect the results and whether any measures were taken to mitigate potential biases.**

**Answer:** We acknowledge that using different sequencing methods (paired-end for 38 samples and single-end for 6) could introduce biases in the results. To minimize these effects, we applied standardized bioinformatics pipelines and normalized the data before analysis. Additionally, we will include a more detailed discussion of these methodological differences and their potential impact on the results to enhance transparency.

**Please see lines 145-154:** In the initial stages of the pipeline, paired-end samples and single-end samples were processed separately. Thus, the samples were divided into two groups, going through all data quality control procedures and the construction of the ASVs (Amplicon Sequence Variants) tables independently. Once we obtained the ASV tables, they were merged into a single ASV table, which was then used in the subsequent stages of the pipeline. In an attempt to obtain the highest possible percentage of concatenated reads (in the case of paired-end sequencing), different tests were carried out, altering the size of the overlap required between the pairs (we varied the size of the overlap from 1 to 20 and, in the end, used 12) to merge the reads. In this way, the percentages achieved are as high as possible.

**3. The authors should consider using more robust statistical techniques for microbiome data analysis, such as PERMANOVA or ANOSIM for beta diversity comparisons.**

**Answer:** We agree that tools such as PERMANOVA and ANOSIM could strengthen beta diversity analyses. Although PERMANOVA has been used in some analyses, we will add a detailed description of its application and justification for choosing the method, as well as ANOSIM to the microbiota analyses.

**Please see lines 260: Fig 4. Analysis of the fecal microbiota's beta diversity according to postmenopause time.**

**Please see lines 263: a) Bray-Curtis distance using the ANOSIM test**

**Please see lines 268: b) Bray-Curtis distance using the PERMANOVA test**

**4. The study focuses primarily on taxonomic composition but lacks insight into the functional implications of the observed microbiome differences. The authors should consider incorporating predictive functional profiling tools to provide more context to their findings.**

**Answer:** Thank you for your suggestion regarding functional analysis. While our primary focus was taxonomic composition, we agree that predictive functional profiling tools like PICRUSt or Tax4Fun could provide deeper insights. We plan to incorporate these analyses in future studies and will acknowledge this limitation in the present work.

**Please see lines 521-539:** Our research has significant limitations that must be reported when studying gut microbiota. We recognize the limitation of a smaller sample size (n=10) in the postmenopausal group for more than 10 years. The small sample size reduces the statistical power to identify subtle differences in microbial composition. Future studies with more participants in this category will be essential to validate our initial observations and give greater robustness to the conclusions. It was not possible to investigate and monitor dietary habits and practice of physical activities. The participants reported using different classes of medication, except antibiotics. We cannot rule out the possibility that these factors may influence the composition of these participants' microbiota. Future studies should be carried out to control these factors in this cohort, and predictive functional analysis should also be included to strengthen the characterization of the intestinal microbiota of Brazilian women.

The strengths of our study involve the participation of a cohort of postmenopausal women whose age range coincides with significant hormonal and metabolic changes primarily related to essential modifications in the intestinal microbiota. Our study contributes to the characterization of this microbiota in middle-aged Brazilian women, showing their resilience, which may open the way for individualized and personalized treatments for conditions that affect women at this stage of life. In addition, we characterized the ten most abundant organisms in this population, which could indicate new actions to prevent postmenopausal health conditions.

**Minor:**

**1. Authors need to include a more detailed description of the participants' demographic and clinical characteristics in the results section, possibly as a table.**

**Answer:** To better contextualize the analysis, we will add a detailed table in the results section containing the demographic and clinical characteristics of the participants, such as age, body mass index (BMI), time since menopause, and medication use. The requested data has been added to Table 1. A table with detailed medications is provided as supplemental material (S1 Table).

**Please see lines 176-182 of Table 1, which shows the Sociodemographic and health characteristics of the women participating in this study.**

**Please see Supplementary material: S1 Table. Medications used by participants in this study.**

**2. Authors need to provide more information on the DNA extraction and library preparation protocols, including any quality control measures.**

**Answer:** We will include a more detailed description of the DNA extraction and library preparation protocols, including quality control methods, to ensure greater transparency in the techniques used.

**Please see lines 114-121:** Bacterial genomic DNA was extracted using 15mg of feces, following the International Human Microbiome Standards (IHMS) recommendations, specifically protocol H (29). Briefly, the extraction process used 15mg of feces, which proceeded with chemical and mechanical lysis steps using guanidine isothiocyanate, N-lauryl Sarcosine, glass beads, and vortex processes. The protocol used Polyvinylpolypyrrolidone and TENP (Tris-HCL buffer, EDTA, NaCl, and PVPP) between centrifugation and washing steps. Finally, the DNA was precipitated with Isopropanol and treated with 10mg/µL of Rnase (Sigma). The DNA was resuspended in 200µL of RNAse-free water and stored at 4°C.

**Please see lines 127-142:** The primers are complementary to the target and to the Illumina adapter, which allows a second PCR by adding the indexing sequences (31). Two µl of DNA were used for each sample as a template in the first PCR reaction. Platinum Taq (Invitrogen, USA) was used under the following conditions: 95 °C for 5 min, 25 cycles of 95 °C for 45s, 55 °C for 30s and 72 °C for 45s, and a final extension of 72 °C for 2 min for the first PCR. For the second PCR, the conditions were 95 °C for 5 min, 10 cycles of 95 °C for 45s, 66 °C for 30s and 72 °C for 45s, and a final extension of 72 °C for 2 min. The final reactions were purified using Neobeads ® (Sera-Mag ™ based magnetic beads) and an equivalent volume of each sample was added to the sequencing pool. A negative control was used for each PCR and the reactions were carried out in triplicate. The final DNA concentration of the library pool was estimated using Picogreen dsDNA (Invitrogen, USA), and diluted for qPCR using the Collibri™ Library Quantification Kit (Invitrogen, USA) already optimized for Illumina libraries. The sequencing pool was adjusted to a final concentration of 11 pM. The remaining six samples were sequenced by *Proteimax Biotecnologia Ltda* (São Paulo, Brazil), which used the Illumina Nextera XT DNA library prep kit for the libraries and the primer 314F (CCTACGGGNGGCWGCAG) on the Illumina NovaSeq6000 (2x250 bp) (*single-end*) (32).

**3. Authors need to include a brief explanation of the chosen alpha and beta diversity metrics for readers less familiar with microbiome analyses.**

**Answer:** To help readers less familiar with microbiome analysis understand, we will add brief explanations of the chosen alpha and beta diversity metrics, highlighting their relevance to the study.

**Please see lines 419-427:** Alpha and beta diversity analyses are essential tools for understanding the "invisible life" in our bodies. Alpha diversity analyses how many different species (or types of microorganisms) exist in a single place, such as a person's intestine (34,36,57). It helps to understand whether that environment has a large variety or is dominated by a few species. Beta diversity, on the other hand, assesses the differences between two environments or groups, such as the intestines of healthy people compared to those of people with some disease (35,38). Together, they allow inferences to be made: How much variety we have in the intestine (or other places) and how the microbiota of one person or group compares to another.

**4. Authors need to consider adding a correlation analysis between specific bacterial taxa and relevant clinical parameters (e.g., time since menopause, age, BMI) to provide more context to the findings.**

**Answer:** We agree that comparing specific bacterial rates and clinical parameters, such as time since menopause, age, and BMI, could enrich the interpretations. We will add these analyses and discuss their implications in the context of our results.

**Please see Supplementary material: S2 Table.** **Correlation analysis between the ten most abundant bacterial phyla, families and genera with clinical parameters .**

We believe we have answered all the reviewer's concerns. The changes have greatly improved the final version of the manuscript, for which we thank our colleagues' scientific assistance. We hope that our manuscript, in its current form, is suited for publication in Plos One. Please advise us if any other changes are necessary.

Cordially,

Prof. Dr. Renata Guerra-Sá

(Corresponding Author)

Laboratory of Biochemistry and Molecular Biology (LBBM), Department of Biological Sciences, Institute of Exact and Biological Sciences, Federal University of Ouro Preto (UFOP). Campus Universitário s/n, Morro do Cruzeiro, Ouro Preto, MG, Brazil.

Postal code: 35400-000. E-mail: rguerra@ufop.edu.br
