# Supplementary material for: Microbiota and Postmenopause: The resilience of intestinal bacteria in the face of female hormonal aging
Source: PLoS One. 2025 Jun 18;20(6):e0324712. doi: 10.1371/journal.pone.0324712 (PMC12176181; doi:10.1371/journal.pone.0324712)
Supplement: Attachment — Submitted filename: Response_to_Reviewers_auresp_2.docx [file pone.0324712.s005.docx]

**Dear Dr. Sayed Haidar Abbas Raza**

Regarding the manuscript [PONE-D-24-55161] - [EMID:5576289f144cdfbc] entitled "Microbiota and Postmenopause: The Resilience of Intestinal Bacteria in the Face of Female Hormonal Aging," we have carefully reviewed and revised the manuscript according to the reviewers' suggestions. Below, we address each point raised, indicating where changes were made in the text. We want to take this opportunity to sincerely thank the reviewers for their thorough and insightful feedback, which greatly facilitated our revision process and undoubtedly enhanced the quality of our manuscript.

**Journal Requirements:**

1. **Please review your reference list to ensure that it is complete and correct. If you have cited papers that have been retracted, please include the rationale for doing so in the manuscript text, or remove these references and replace them with relevant current references. Any changes to the reference list should be mentioned in the rebuttal letter that accompanies your revised manuscript. If you need to cite a retracted article, indicate the article's retracted status in the References list and also include a citation and full reference for the retraction notice.**

The list of references has been fully revised as requested. All references have been supplied correctly. We do not cite references that have been retracted.

**ANSWER TO REVIEWER n°2**

1. **Certain paragraphs reiterate similar points without adding new insights.**

**Answer:** We appreciate your comment and have carefully revised the manuscript to minimize similar points and make it more straightforward. As it was not specified, we marked them in the text, and you can check the changes in the paragraphs and lines where we removed and added them.

2. **The readers could benefit from a table summarizing participants' BMI, comorbidities, and medication use.**

**Answer:** Thank you for your suggestion. The information requested on the participants' BMI, comorbidities, and medication use has been added to Table 1.

**Please see lines 191- 196:** Table 1. Sociodemographic and health characteristics of the women participating in this study.

3. **The authors are suggested to add the different biasis with the different sequencing method.**

**Answer:** We acknowledge that using different sequencing methods (paired-end for 38 samples and single-end for 6) could introduce biases in the results. We applied standardized bioinformatics pipelines to minimize these effects and normalized the data before analysis. We have included this information as a limitation of our study, clarifying the potential impact on the results by making the methodology bias transparent to readers.

**Please see lines 520- 523:** Different sequencing methods (paired-end for 38 samples and single-end for 6) could bias the results. However, to minimize these effects, we applied standardized bioinformatics pipelines and normalized the data before analysis to make the reads comparable without prejudicing subsequent analyses.

4. **Readers would be also benefitted from mentioning of functional implications of microbiome changes.**

**Answer:** Thank you for your suggestion. Our research group would like to highlight a few important points based on a recent study done by Turjeman and collaborators (Turjeman S, Rozera T, Elinav E, Ianiro G, Koren O. From big data and experimental models to clinical trials: Iterative strategies in microbiome research. Cell. 2025 Mar 6;188(5):1178-1197. doi:10.1016/j.cell.2025.01.038. PMID: 40054445.)

Studies involving the intestinal microbiome face many challenges. One of the most significant challenges is the functional implications of changes in intestinal bacterial communities. The authors discuss at length that the field of research studying the microbiome and human diseases has been based mainly on correlative studies, and determining, for example, whether dysbiosis is a cause or consequence of diseases and their adjacent mechanisms is still the biggest challenge.

Finding correlations does not mean causality, as described by the authors. Extensive studies that have demonstrated some relationship between microbial modifications and the occurrence of diseases such as inflammatory bowel disease, colorectal cancer, diabetes, and host metabolism have yet to provide direct proof of this causal relationship between the microbiome and clinical outcomes. Even with advanced modeling and integrated multi-omics, this correlation and validation remain *in silico*.

Our research group agrees that for more compelling statements about the functional implications of modifications to the human microbiome, it is necessary to employ an iterative approach combining in silico, in vitro, ex vivo, and in vivo data, as suggested by the aforementioned work. For this reason, we have chosen not to speculate on functional mechanisms in this work. We intend to apply this iterative approach to the continuity of our future research with this cohort.

We believe we have answered all the reviewer's concerns. The changes have greatly improved the final version of the manuscript, for which we thank our colleagues' scientific assistance. In its current form, we hope that our manuscript is suited for publication in Plos One. Please advise us if any other changes are necessary.

Cordially,

Prof. Dr. Renata Guerra-Sá

(Corresponding Author)

Laboratory of Biochemistry and Molecular Biology (LBBM), Department of Biological Sciences, Institute of Exact and Biological Sciences, Federal University of Ouro Preto (UFOP). Campus Universitário s/n, Morro do Cruzeiro, Ouro Preto, MG, Brazil.

Postal code: 35400-000. E-mail: rguerra@ufop.edu.br
